# Gingival Overgrowth in an Adult Male Patient

**DOI:** 10.7759/cureus.22572

**Published:** 2022-02-24

**Authors:** Roopali Mahajan, Faizan Alawi, Katherine France

**Affiliations:** 1 Oral Medicine, University of Pennsylvania School of Dental Medicine, Philadelphia, USA; 2 Basic and Translational Sciences, University of Pennsylvania School of Dental Medicine, Philadelphia, USA

**Keywords:** reactive benign lesion, gingival overgrowth, exophytic nodule, bone loss, pyogenic granuloma

## Abstract

Pyogenic granuloma (PG) is an oral reactive inflammatory hyperplasia of connective tissue that can occur in response to hormonal changes and local irritation such as calculus, fractured teeth, rough dental restorations, and foreign materials. It is nonneoplastic and predominant in the second decade of life in young adult females. The most common site of involvement is the gingiva. Lesions are more common in the maxillary than mandibular gingiva and mainly occur on the facial or buccal aspect. Pyogenic granuloma rarely grows more than 2 cm in diameter and is rarely associated with bone loss. This article presents a rare case of an abnormally large pyogenic granuloma affecting both the labial and palatal gingiva sustaining occlusal trauma due to its size and associated with severe alveolar bone loss that was managed successfully with surgical excision in a 40-year-old male.

## Introduction

Inflammatory hyperplasia is an umbrella term that describes reactive oral mucosa consisting of enlarged fibrous and granulation tissues, including pyogenic granuloma (PG) [1]. Pyogenic granuloma is nonneoplastic reactive hyperplasia that develops in the context of various local and systemic factors [2,3]. It is predominant in females during the second decade of life with a female to male ratio of 2:1 [3,4]. While the term 'pyogenic granuloma' would suggest a granular cell reaction associated with pus formation, it is, in fact, a lesion that is reactive and spurred by systemic (hormonal changes) or local (poor oral hygiene, broken teeth, uneven dental fillings, and impacted or retained foreign materials) irritants [4]. Additionally, on histopathology, PG represents connective tissue enlargement rather than the formation of a granuloma [4]. The most common triggering factor is poor oral hygiene with 75% of all cases showing tartar deposits or foreign substances in the associated gingival crevice [3]. A further 75% of PG lesions are seen on the gingiva. Pyogenic granuloma can also affect the lips, mucosa, tongue, and other areas, but is seen rarely in these locations [5,6]. Clinically, PG appears as a uniform, lobulated, exophytic growth with a pedunculated or sessile base [3,5]. The lesion is often asymptomatic, painless, and develops slowly, although rapid growth can occur in some cases [3,5]. Lesions are usually hemorrhagic, often abundantly [5]. The color of the lesion ranges from pink to red to purple with newer, more vascular lesions presenting with deeper visible pigmentation [3]. As lesions mature, the vascularity decreases, and the clinical appearance becomes thickened, dense and pink in color [4,5].

While the existence of PG is well documented in the literature, published cases tend to adhere to the above common presentations, with abnormal manifestations rarely detailed. To add evidence of the clinical variation in PG, this article describes an unusual case report of a 40-year-old male with an abnormally large pyogenic granuloma extending from the labial to the palatal side of the upper left anterior gingiva that was associated with occlusal trauma and significant alveolar bone loss mimicking malignancy, which was managed by surgical excision.

## Case presentation

**Figure 1:**
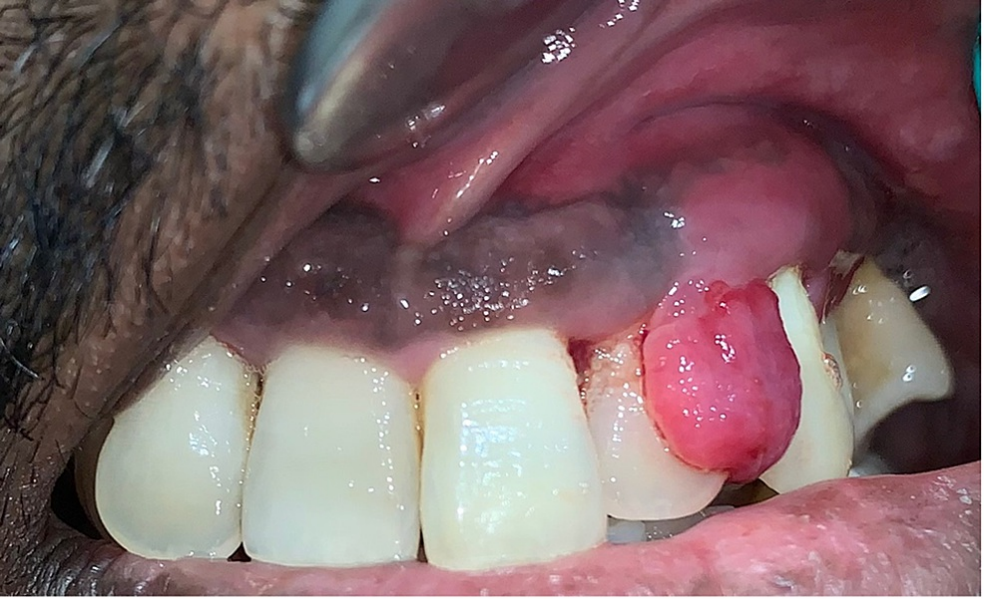
The growth extending from the distal aspect of the lateral incisor to the mesial aspect of the canine

A 40-year-old systemically healthy male patient presented to the comprehensive care clinic at Penn Dental Medicine for the evaluation of an intraoral growth in the mouth involving the upper left anterior gingiva. The patient had noticed a small painless growth on the palatal gingiva that began about 11 months ago. He reported a gradual increase in the size of the growth, which with time led to discomfort during eating and talking. He described that by the time of presentation the size of the growth interfered with these functions and was causing changes in his bite. The patient also reported evolution in location i.e. the growth enlarged from the palatal to facial side interdentally and caused an increase in the space between the left maxillary lateral incisor and canine. He also endorsed bleeding from the gums both spontaneously and on brushing which was especially frequent in this area. He denied a history of fever, fatigue, tiredness, loss of appetite, paresthesia, and weight loss as well as other systemic symptoms. He denied past medical conditions and current medications or allergies. Social history was positive for a smoking habit of 10 cigarettes per day for 20 to 25 years that he quit two years ago. Extraoral examination did not reveal any significant findings. No evidence of lymphadenopathy was seen. On intraoral examination, a bilobular gingival overgrowth extending palate-facially from the left lateral incisor to canine was noticed. Facially, the growth was round, soft, smooth, pedunculated, dark pink in color, around 1 cm x1 cm in size, and extended from the distal aspect of the lateral incisor to the mesial aspect of the canine (Figure 1).

**Figure 2:**
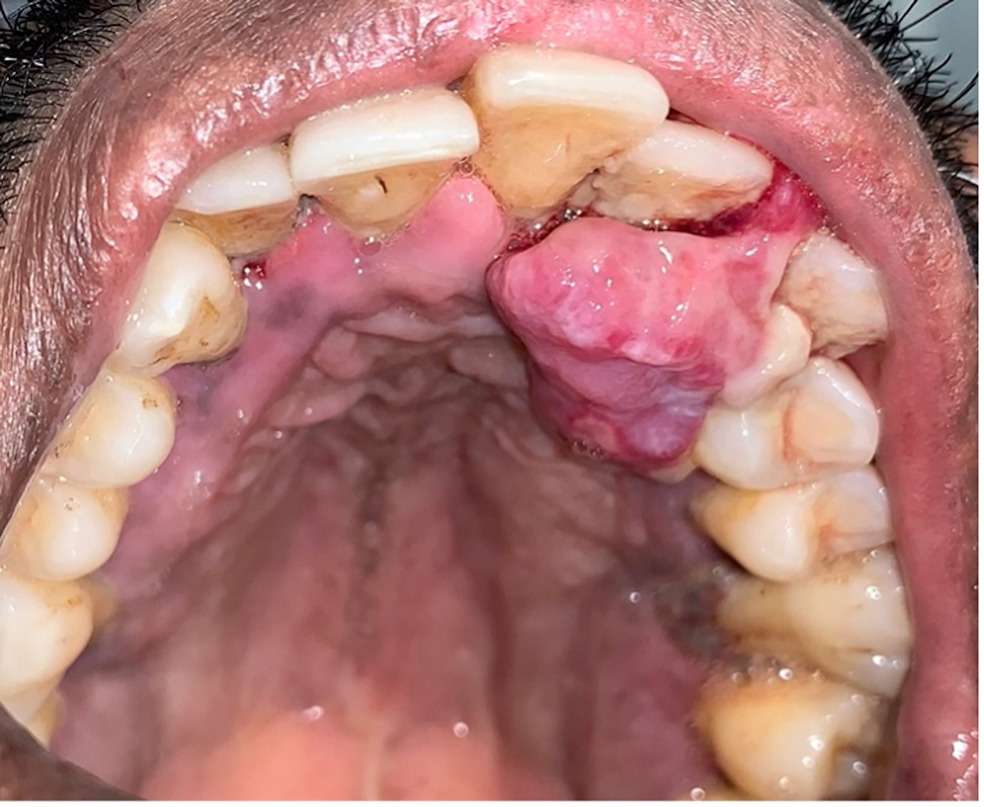
The palatal lesion is observed to be coral pink in color with multiple intermixed red and white areas across the extent of the surface

Palatally, the growth was rough, pedunculated, around 3 cm x3 cm in size, and extended from the distal aspect of the central incisor to the mesial aspect of the premolar with poorly defined borders. The palatal lesion was coral pink in color with multiple intermixed red and white areas across the extent of the surface (Figure 2). It was firm but not indurated and exhibited minimal tenderness to palpation. Indentations along the palatal portion of the lesion were seen with surface ulceration that on occlusal analysis corresponded to constant trauma from lower dentition. There was also evidence of food impaction within the grooves of the lesion’s palatal aspect. Both the upper permanent lateral incisor and canine exhibited mobility of grade two. Pathological migration of the canine labially was also present. The patient’s oral hygiene was poor with significant generalized supra- and sub-gingival plaque, calculus and debris, gingival erythema and edema, coronal staining, and halitosis. Based on history and clinical presentation, a clinical diagnosis of localized gingival hyperplasia and generalized stage 2 grade B periodontitis was made [7].

**Figure 3:**
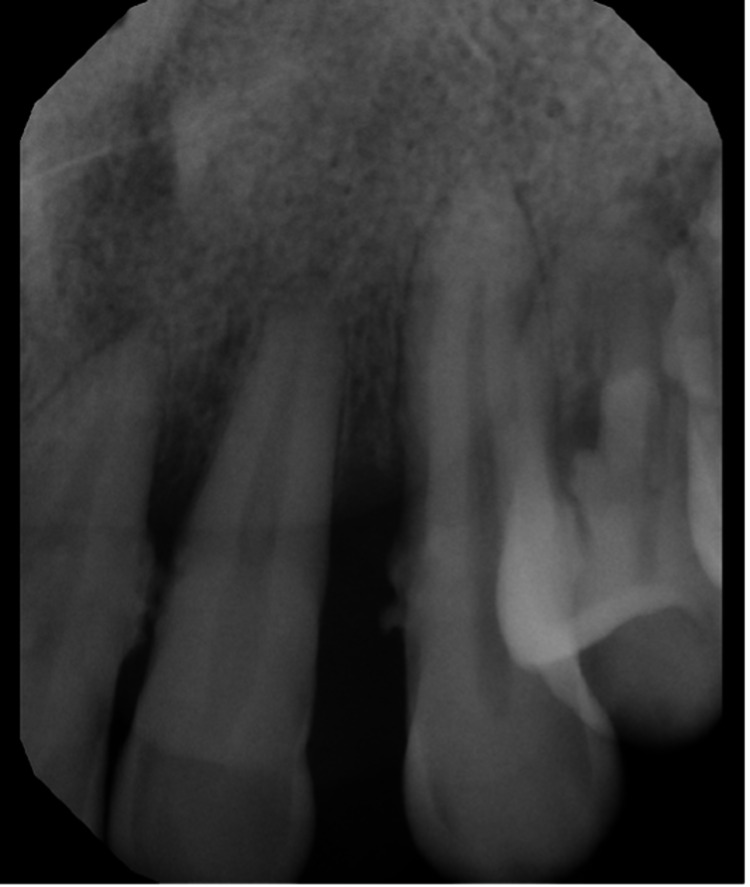
Intraoral periapical radiograph of the upper left lateral incisor and the canine region

An intraoral periapical radiograph of the upper left lateral incisor and the canine region was taken at presentation, which revealed a generalized widening of the periodontal ligament spaces and severe horizontal alveolar bone loss from the alveolar crest to the middle one-third of root (Figure 3). The bone loss was more severe in the affected maxillary left anterior region than in other areas and corresponded to the location and size of the lesion. The roots of the involved teeth did not show any evidence of bone resorption and the crowns of the teeth showed no caries clinically or radiographically.

The differential diagnosis for the patient included PG, granulation tissue hyperplasia, peripheral giant cell granuloma, peripheral ossifying fibroma, hemangioma, oral squamous cell carcinoma, and metastatic cancer. Given the clinical variability of each of these lesions, the determination of final diagnosis is primarily based on biopsy and histologic findings. Conventional hyperplastic gingival inflammation is difficult to differentiate from pyogenic granuloma histopathologically, and in such cases, diagnosis is based on the clinical description of the lesion. PG shows endothelial cell proliferation with inflammatory cell infiltration. Peripheral giant cell granuloma only occurs on the gingiva is diagnosed based on multinucleated giant cells and the presence of extracellular hemorrhage on histopathology. Peripheral ossifying fibroma (POF) and peripheral odontogenic fibroma also occur exclusively on the gingiva. They are characterized by cellular fibrous proliferations with either metaplastic bone (ossifying) or epithelial rests (odontogenic). Clinically, it is very difficult to differentiate PG from POF as the former becomes more fibrotic as the age of lesion advances. Radiographically, POF rarely shows bony changes which include cupping defect of the alveolar bone around the roots, occasional tooth displacement, and diffuse multiple radiopacities representing calcifications. However, in long-standing cases, POF may show significant bone destruction. Moreover, POF shows high cellularity with calcified material derived from the periodontal ligament or periosteum, balanced with less vascularity, and peripheral odontogenic fibroma shows dispersed odontogenic epithelium within the stroma histologically. Hemangioma shows endothelial cell proliferation without inflammatory cell infiltrate. While rare, metastatic tumors can present in various locations of the mouth including the gingiva and may appear clinically similar to inflammatory hyperplastic growths such as PG or other entities in the differential diagnosis. On excision, however, these are easily differentiated and histologically represent the primary tumor.

**Figure 4:**
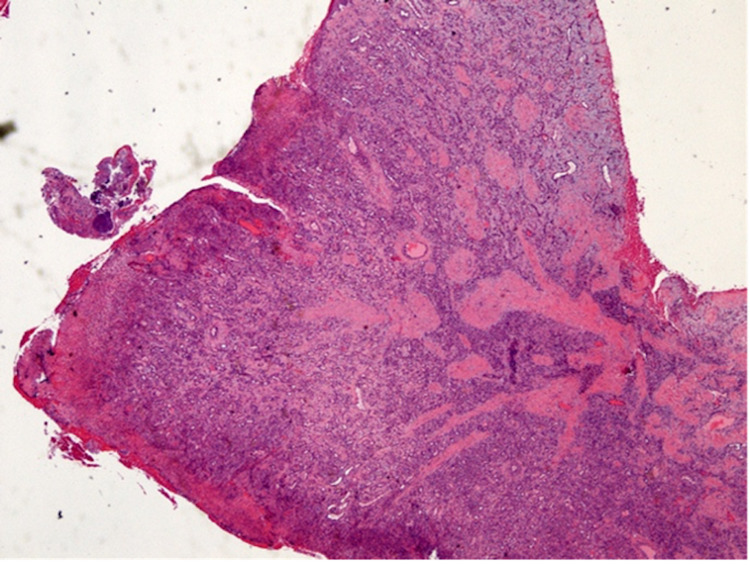
Histopathological finding showing focally ulcerated stratified squamous epithelium covering a core of loose and edematous fibrous connective tissue

**Figure 5:**
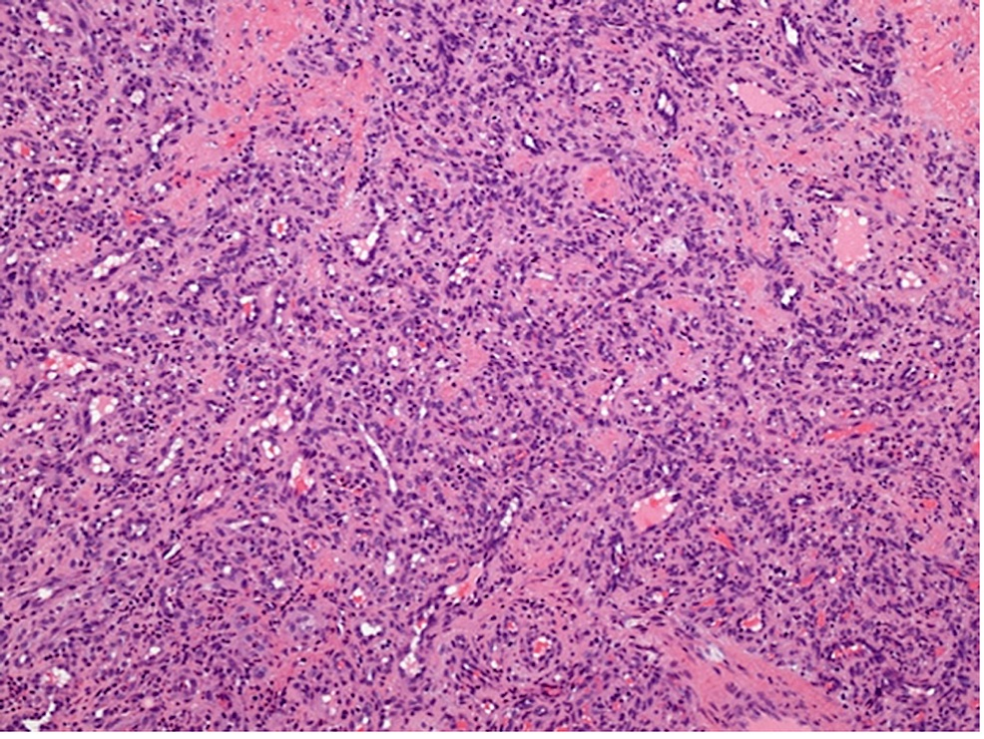
Histopathological finding showing the stroma containing numerous endothelial-lined blood-filled capillaries and a dense infiltrate of lymphocytes, plasma cells and neutrophils

Due to discomfort in eating and talking, interference with occlusion, and food impaction, gross excision of the lesion and histopathologic evaluation was planned despite the relatively large size and poorly defined margins. Given the severe bone loss and bleeding tendency as well as the spread of inflammation into surrounding tissue, this was determined to be the most thorough path to definitive diagnosis and appropriate to the patient’s complaints. The patient agreed to this plan and was pleased with a solution that would restore his comfort and occlusion. Scalpel biopsy was planned due to the area affected and was chosen over laser and cryosurgery secondary to considerations of access and financial burden. An excisional biopsy was completed under local anaesthesia. The lesion was removed completely with curettage and irrigation to remove granulation tissue. Hemostasis was achieved with silver nitrate cautery and pressure; the initial clot was confirmed before the patient was dismissed. Despite the heavily hemorrhagic nature of the lesion, bleeding was not excessive and was controlled during and after the procedure easily with local measures. Histopathological findings (Figures 4, 5) showed focally ulcerated stratified squamous epithelium covering a core of loose and edematous fibrous connective tissue. The stroma contained numerous endothelial-lined blood-filled capillaries and a dense infiltrate of lymphocytes, plasma cells and neutrophils. The ulcer bed consisted of a fibrin network entrapping viable and necrotic neutrophils and lymphocytes.

The above histopathological features were consistent with pyogenic granuloma. After one month, the patient healed well from the procedure, was informed of the diagnosis, and was counselled on the importance of maintaining meticulous oral hygiene and regular follow-ups to avoid further bone loss, recurrence, and secondary complications from periodontal disease. He was strongly recommended to seek definitive dental treatment given his compromised periodontal status. However, to date (six months after biopsy), he has failed to attend additional appointments to start this process. Our interim communication with him has confirmed that he is well without current complaint in the area of the excised lesion.

## Discussion

Pyogenic granuloma is non-neoplastic inflammatory hyperplasia [8]. It occurs more commonly in females than males, with a ratio of 2:1 [9]. In a review study, PG incidence was found to peak in the second decade and develop in 22% of young patients [9]. Contrary to these averages, our case presents a PG that occurred in a male in his early 40s. Local irritants commonly precipitate the development of these lesions and may include defective restorations, plaque and calculus, and accumulation of foreign materials [8]. The patient presented here exhibited severe plaque and calculus build up as well as generalized periodontal disease, which may have contributed to lesion development. Clinically, PG presents as a painless, smooth, or lobulated mass. Its highly vascular structure causes frequent hemorrhage in a majority of cases, including our patient [10]. The color of the lesion also varies and is dependent on the extent of underlying vascularity in relation to the clinical course [11]. Sometimes, as in the reported case, the surface may be covered by a pseudo-membrane due to secondary ulcerations [10]. Radiographic findings in PG are usually absent [8]. Occasionally, PG is associated with bone destruction [12]. Goodman-Topper et al. reported a significant bone loss associated with PG in the upper teeth of a male child patient [13]. Another case of PG associated with a cupping defect of alveolar bone between the primary molar and first permanent molar in a very young girl was documented by Shenoy et al. [14]. Some additional studies by Singh et al. and Saikhedkar et al. have also reported PG associated with mild bone loss in adult patients in the upper front region [15,16]. Furthermore, Angelopoulos also concluded that sometimes long-standing gingival pyogenic granulomas cause localized alveolar bone resorption leading to localized periodontitis [17]. In the present case, there was a severe generalized alveolar bone loss which was particularly advanced bone surrounding the PG. The pattern of bone destruction in our case could be caused by the pressure from the formation of the PG but also could represent local advancement of his overall severe periodontal disease, possibly due to local irritants or inflammation.

Our patient had sought dental treatment very intermittently, limiting our ability to compare radiographic findings over time. His inconsistent follow up also helps to explain the large size of the lesion on evaluation. Pyogenic granuloma can be categorized into three distinct stages, namely, (i) cellular phase, (ii) capillary phase/vascular phase, and (iii) involution phase [11]. Depending on the different stages of PG, the common differential diagnosis includes peripheral giant cell granuloma, peripheral ossifying fibroma, oral squamous cell carcinoma, metastatic cancer, hemangioma, and granulation tissue hyperplasia [8,11,18,19]. Definitive diagnosis of PG is based on biopsy and histopathologic examination [8]. Microscopically, PG shows proliferation of excess granulation tissue covered by a mix of atrophic and hyperplastic epithelium with variable surface ulceration and intralesional fibrinous exudates [8]. Another important histopathologic feature of PG is the presence of vascular spaces lined by endothelium with proliferating fibroblasts and endothelial cells. Infiltration of mixed inflammatory cells is also seen [8].

Surgical excision of the lesion followed by curettage of the underlying tissue is the first line of treatment for gingival pyogenic granuloma [3]. The other treatment options include cryotherapy, electrocautery, sclerotherapy with sodium tetra decyl sulfate and monoethanolamine oleate ligation, pure ethyl alcohol injection dye, neodymium-doped yttrium aluminum garnet (Nd:YAG) and carbon dioxide (CO2) laser photocoagulation, and intralesional corticosteroids [20,21].

The benefits of using laser surgery over traditional options are less chances of infection, reduced bleeding, precise cutting with good judgement of cutting depth, lesser need for antibiotics and analgesics post operatively, no need for suturing, reduced pain during and after the procedure, faster wound healing, less chances of recurrence and scarring [21]. Despite these advantages, lasers are not extensively used because they are not cost effective due to high upfront and operational costs. Moreover, skills and technical knowledge are of paramount importance for dentists or dental specialists working with lasers [21].

Cryosurgery involves localized and targeted destruction of abnormal tissue by the application of cold substances like liquid nitrogen spray and gases such as nitrous oxide, carbon dioxide, and freons [22]. Cryotherapy causes less trauma, discomfort, and pain, reduces bleeding and scarring, reduces risk of infection, is more localized in action, preserves the mineralized component of bone, and is easy to employ [22]. It is most useful in patients in whom surgery is contraindicated due to factors like age or complicated medical history. The limitations of cold therapy include the possible disproportionate degree of swelling and lack of precision in terms of depth and area of freezing. Cryotherapy also requires advanced operator skills and experience [22].

Given the symptoms present in this case, and consistent with clinical guidelines, we elected to treat the patient definitively and immediately via excision and curettage. Any foreign body, calculus, a defective restoration, or other local contributing factors should also be removed, which was recommended for this patient but which he has not yet completed [3]. Intralesional corticosteroids, lasers, and cryosurgery are other possible treatments [2]. In this case, in line with recommendations and despite the large size, the lesion was successfully excised, and the underlying bone was curetted in hopes of minimizing recurrence. Given the advanced periodontal disease and heavy plaque and calculus build up, the patient was strongly recommended to seek further dental treatment both for overall health and to control these contributing factors. Pyogenic granuloma has a recurrence rate of 16% mostly on the gingiva and is due to incomplete removal, failure to remove the etiological factors or re-injury to the affected gingival area [2,9]. This patient will be followed-up as possible during and after comprehensive dental treatment to monitor any recurrence in this or other areas and for stabilization of his oral condition.

## Conclusions

Pyogenic granuloma is a commonly occurring benign inflammatory lesion seen in the oral cavity. It is mostly associated with local irritating factors. It may occasionally have serious outcomes due to its structural attributes and easy bleeding. It is paramount to recognize this lesion at an initial stage to avoid inadvertent and aggressive treatment and to intervene promptly to prevent recurrence and rule out other causes of localized swelling. Complete removal of gingival overgrowth with curettage reduces the risk of recurrences. Close monitoring and good oral hygiene practices are also required. This case illustrates the possibility of PG to grow to a large size and present with severe bone loss around the teeth as well as the ability of these lesions to interfere with normal function.
